# Pyrimidines: A New Versatile Molecule in the Drug Development Field, Scope, and Future Aspects

**DOI:** 10.3390/ph17101258

**Published:** 2024-09-24

**Authors:** Katharigatta N. Venugopala, Vinuta Kamat

**Affiliations:** 1 Department of Pharmaceutical Sciences, College of Clinical Pharmacy, King Faisal University, Al-Ahsa 31982, Saudi Arabia; 2 Department of Biotechnology and Food Science, Faculty of Applied Sciences, Durban University of Technology, Durban 4001, South Africa; 3 Department of Chemistry, Mangalore University, Mangalagangothri, Mangaluru 574 199, Karnataka, India

**Keywords:** pyrimidines, biologically potent, nitrogen-containing heterocycles, nucleobases

## Abstract

Pyrimidine is a moiety that occurs in living organisms and has a variety of significant biological properties in pharmacology. Due to the easy handling of synthesis, easily available precursor, and less duration for the reaction, for the synthesis, not many technical skills are needed. All these factors attract chemists to focus more on pyrimidines. Apart from the synthesis of biological applications of pyrimidines, medicinal chemists have gathered to explore more pyrimidine scaffolds due to their interesting medicinal properties and easy targeting of various binding sites. This review delves into the diverse biological activities of compounds derived from pyrimidine during the year 2024. We have attempted to explore the growing significance of pyrimidine derivatives and provide a new path for designing new potent molecules.

## 1. Introduction

**Figure 1:**
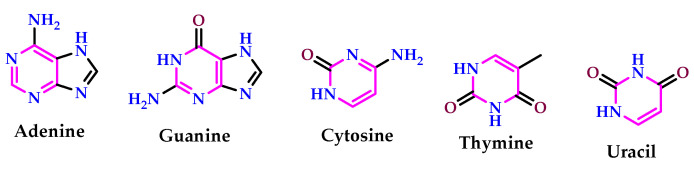
Well-known *N*-containing heterocycles are present in nucleobases.

**Figure 2:**
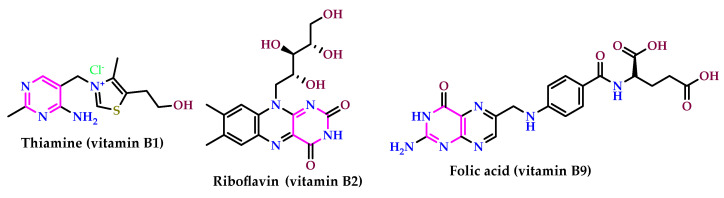
Pyrimidine-containing vitamins.

**Figure 3:**
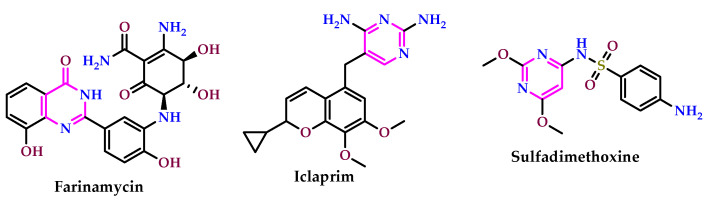
Pyrimidine-containing antibiotics.

**Figure 4:**
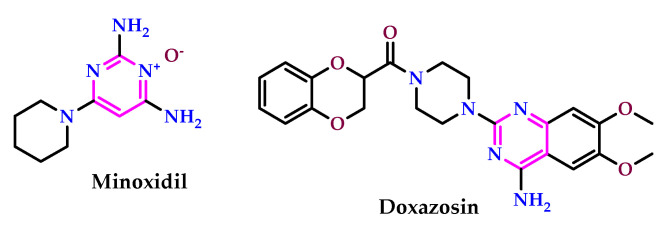
Pyrimidine-containing antihypertensive drugs.

**Figure 5:**
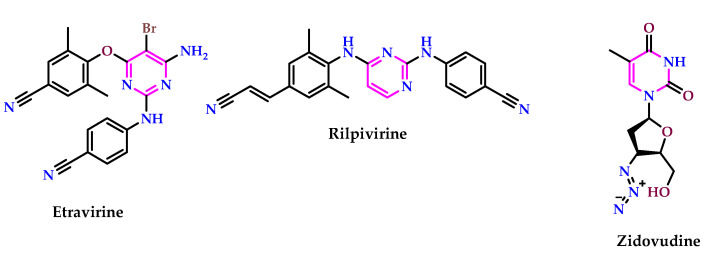
Pyrimidine-containing anti-HIV drugs.

**Figure 6:**
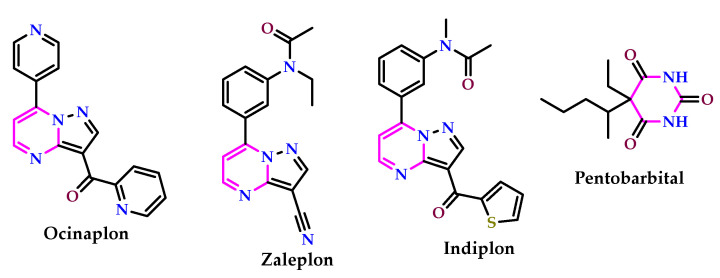
Pyrimidine-containing sedative and hypnotic drugs.

**Figure 7:**
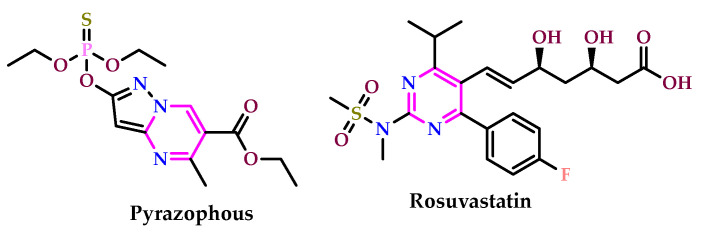
Pyrimidine-containing fungicide and an insecticide and cardiovascular drug.

**Figure 8:**
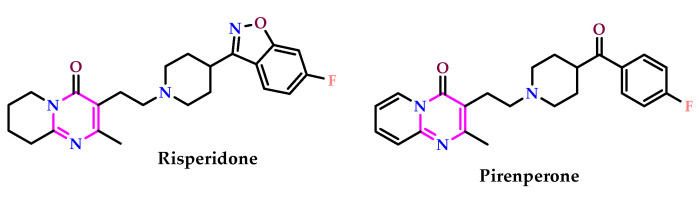
Pyrimidine-containing schizophrenia and bipolar disorder and antipsychotic drugs.

In the living organism, *N*-containing heterocycles are the foremost class, as they consist of the majority of all the parts. Among the *N*-containing heterocycles, pyrimidines are one of the important classes due to biological significance, and these are also found in all living organisms. Pyrimidines are electron-rich heterocycles containing two nitrogens in the ring. Pyrimidine derivatives are present in nucleobases (Figure 1), vitamins (Figure 2), dyes, agrochemicals, antibiotics (Figure 3), etc. Some of the pyrimidine derivatives are used in various medications, e.g., Minoxidil is employed to manage hypertension (Figure 4) and alopecia, Doxazosin is prescribed for hypertension treatment, Etravirine, Rilpivirine, and Zidovudine are used to treat HIV (Figure 5), Zaleplon, Indiplon, and Ocinaplon are used as sedatives and hypnotics (Figure 6), Pyrazophos is used as a fungicide and an insecticide (Figure 7), Rosuvastatin is used in the treatment of cardiovascular disease (Figure 7), Risperidone is used to treat schizophrenia and bipolar disorder (Figure 8), and Pirenperone is an antipsychotic drug (Figure 8). The pyrimidine ring frequently enhances the pharmacokinetic/pharmacodynamic qualities of the medication because of its capacity to interact with a variety of targets by efficiently generating hydrogen bonds and by serving as bioisosteres for phenyl as well as other aromatic π systems [1].

**Scheme 1:**
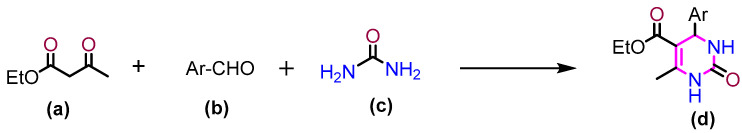
Overall Biginelli reaction synthetic route.

The Biginelli reaction, a well-known multicomponent, one-pot reaction, is used to manufacture pyrimidines. This process, first described by Pietro Biginelli in 1891, condenses three ingredients: urea (compound c), aryl aldehyde (compound b), and ethyl acetoacetate (compound a). This enables the effective production of pyrimidine derivatives. These reactants combine in an RB, where they go through condensation and cyclization processes to produce pyrimidines (compound d). The Biginelli reaction holds great significance in medicinal chemistry because of its ease of use, effectiveness, and capacity to provide diverse pyrimidine-based compounds that exhibit biological activity. This procedure, shown in Scheme 1, demonstrates how a one-pot synthesis can simplify the synthesis of complicated heterocyclic compounds by eliminating the need for several steps and providing a flexible route for the synthesis of pyrimidine derivatives.

**Figure 9:**
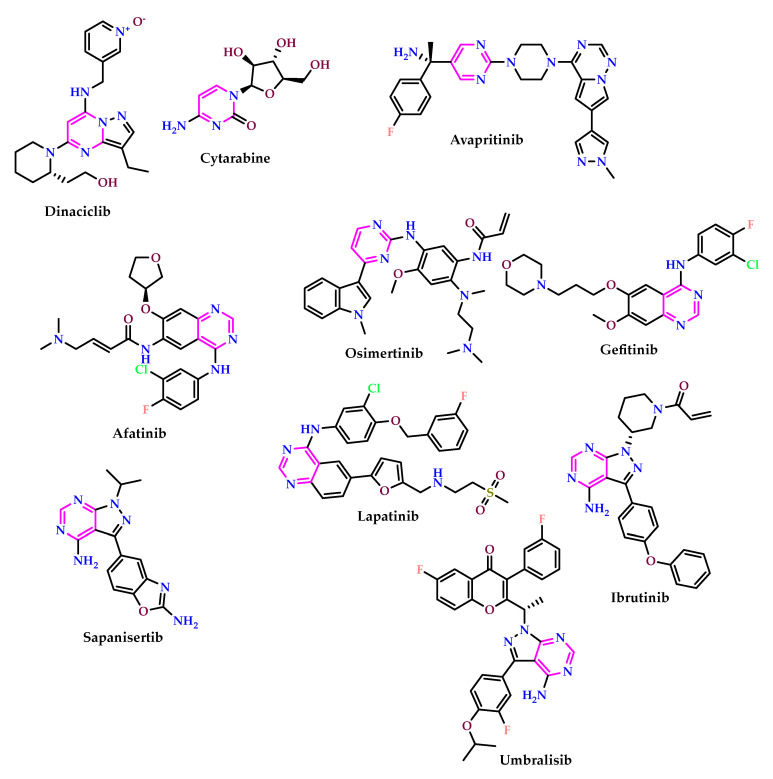
Pyrimidine-containing anticancer drugs.

Various pyrimidine-containing derivatives are used in a variety of cancer treatments, e.g., Dinaciclib is used as CDK, Cytarabine as induction chemotherapy, Avapritinib in the treatment of mastocytosis, Neratinib and Afatinib as a tyrosine kinase inhibitor, osimertinib, Gefitinib as EGFR TKI, Lapatinib as HER2/neu EGFR, Ibrutinib as Bruton’s tyrosine kinase, Sapanisertib as an inhibitor of mTOR, Umbralisib as PI3K-delta and casein kinase CK1-epsilon (Figure 9).

**Figure 10:**
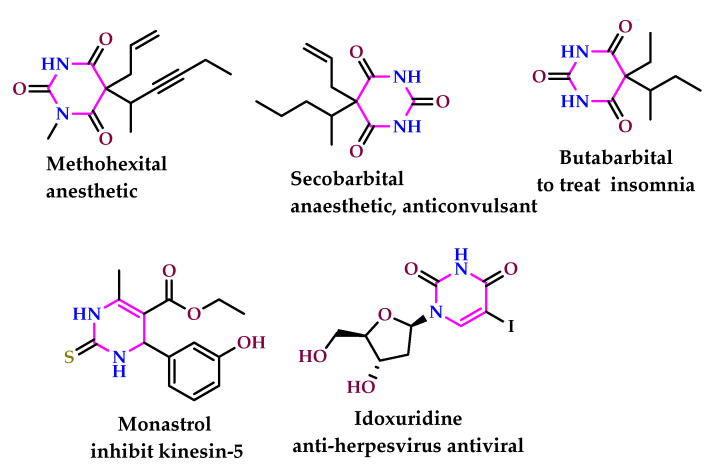
Pyrimidine-containing drugs are used in the market.

Pyrimidine derivatives are well known to possess a variety of biological properties (Figure 10), like antibacterial [2], anti-inflammatory [3,4], antifungal [5], antileishmanial [6], anticancer [7,8], analgesic [9], anticonvulsant [10], antihypertensive [11], insecticidal [12], antidiabetic [13], antiviral [14], anthelmintic [15], antitubercular [16], larvicidal [17,18,19], and antioxidant [20]. Apart from pharmacological applications, pyrimidine derivatives are also found in agrochemicals [21], the petroleum industry [22], corrosion inhibitors [23], fluorescent receptors showing dual signaling mechanisms [24], bioimaging [25], fluorescent pseudomonads [26], photosensitizers [27], Photophysics and Nonlinear Optical Properties [28], dyes [29], electroluminescence [30], Organic Light-Emitting Diodes [31], optoelectronics [32], and organic semiconductors [33]. 

**Figure 11:**
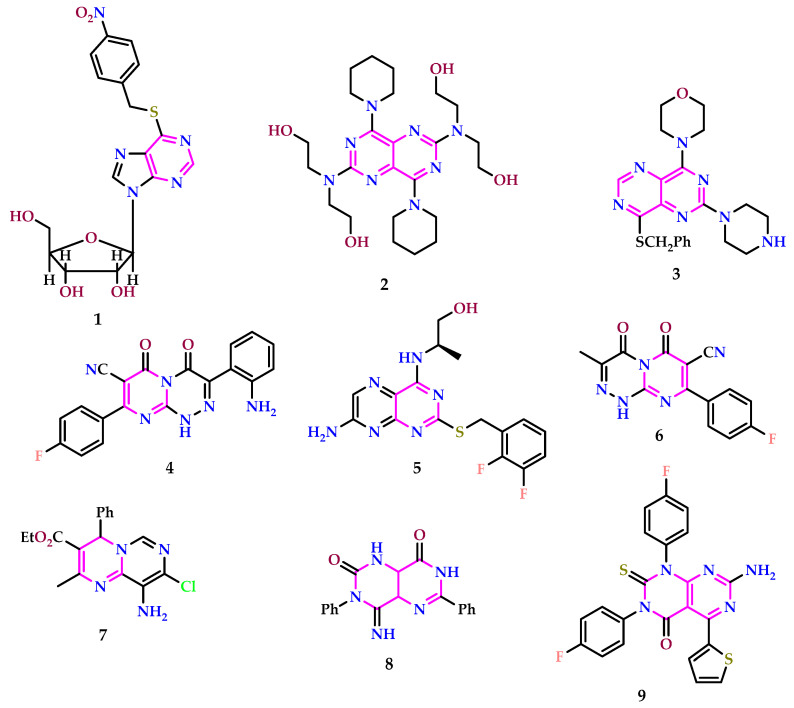
Pyrimidine-containing bioactive molecules reported in the literature.

Because of the extraordinary significance of pyrimidines, a great swath of research efforts has proceeded to study these scaffolds [34] (Figure 11). Apart from its easy accessibility, the pyrimidine skeleton can be readily altered to create structural variation at positions 2, 4, 5, and 6. In this regard, numerous studies have been written that provide a general discussion of the synthesis of pyrimidine analogs and their significance as a class of potentially pharmacologically active compounds [35]. This review concentrates on investigating the pharmacological applications of pyrimidine derivatives and examining their SAR. 

## 2. Pharmacological Properties

In this review, we have focused on various pharmacological properties of pyrimidine and pyrimidine-containing compounds. There have been recent developments in the pyrimidines against various targets, such as EGFR, HDAC, and CDKs. This study aims to explore recent advancements in the anticancer, antimicrobial, antidiabetic, anti-inflammatory, and antioxidant properties of the pyrimidines.

### 2.1. As Anticancer Agents

Tiwari et al. synthesized 27 pyrimidine-triazoles and screened them against MCF-7, MDA-MB453, and MDA-MB231 cell lines; YM155 and menadione were employed as positive controls via the cell viability method. One of the representative compounds, compound **10**, emerged as a better anticancer agent against MDA-MB453, with IC_50_ = 29.1 µM, but compound **11** exhibited better activity against MCF-7 cells, with IC_50_ = 15.3 µM. Both compounds **10** and **11** have the same structures, but different stereochemistry made them drastically different in terms of their anticancer properties [36].


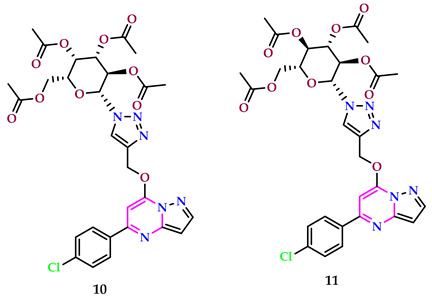


Sabita et al. employed etoposide, a widely recognized chemotherapeutic drug, as a reference point to introduce a new set of pyrimidine-pyrazine-oxazole compounds integrated with chalcone. They then assessed the anticancer potential of these compounds against SiHa, A549, MCF-7, and Colo-205 cell lines using the MTT assay. Most of the examined drugs showed greater activity in comparison to etoposides, based on the data that were acquired. Compounds **12** and **13** exhibited the strongest antitumor action. Compound **12**, featuring a 4-pyridyl group conferred to an unsaturated functional group, demonstrated significant anticancer efficacy, with IC_50_ values given in Table 1. When the 4-pyridyl ring was replaced with a 2-thiazolyl ring in compound **13**, its activity was further improved, with IC_50_ values compared to **12** [37].


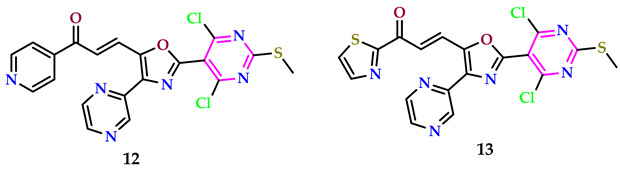


Sivaiah et al. created pyrimidine products as anticancer (against MCF-7, HepG2, and A549 cancer cells) agents using erlotinib as a reference medication; they are powerful dual inhibitors of HDAC and EGFR [38]. Compounds **14** and **15** showed significantly higher efficacy against MCF-7, A549, and HepG2 cell lines, as well as increased safety versus normal WI-38 cells. Compounds **14** and **15** inhibited the EGFR L858R/T790M mutant kinase significantly, with IC_50_ values of 8.43 and 6.91 nM, respectively. Compound **15** exhibited greater inhibitory potency compared to the reference drug SAHA against the analyzed HDAC1, HDAC2, HDAC4, and HDAC6 isoenzymes, with IC_50_ values of 22.73, 20.08, 3100, and 3.71 nM, respectively.

Compound **15**, featuring a nitro-substituted piperidine linked to pyrimidine, stands out as the most powerful within the sequences. It displays IC_50_ values of 2.74, 4.92, and 1.96 μM against the MCF-7, HepG2, and A549 cell lines, respectively. Compound **14**, with a fluorine-substituted piperidine linked to pyrimidine, has IC_50_ values of 3.01, 5.88, and 2.81 μM against the MCF-7, HepG2, and A549 cell lines, respectively. Both molecules were more powerful than the clinically utilized erlotinib, with IC_50_ values of 19.51, 23.61, and 15.83 μM, respectively.


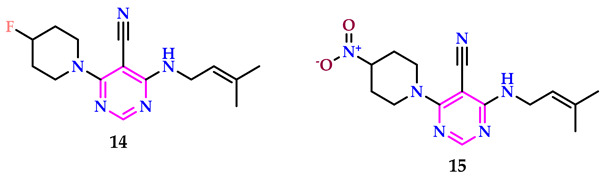


Vemuluri et al. created aryl amide conjugates of thiazole-benzothiazole-pyrimidines and tested them against MCF-7, A549, Colo-205, and A2780 cells, with etoposide as the reference. The results of the in vitro assays indicated that compounds **16**, **17**, **18**, and **19** exhibited greater efficacies compared to the standard agent. Among these compounds, **16** demonstrated particularly potent anticancer activity. Specifically, compound **16**, containing an electron-rich group (3,4,5-tri methoxy), displayed superior activity compared to the standard across all cell lines tested (MCF-7 = 0.09 ± 0.0085, 2.19 ± 1.87 µM; A549 = 0.03 ± 0.0056, 3.34 ± 0.152 µM; Colo-205 = 0.01 ± 0.074, 0.17 ± 0.034 µM; and A2780 = 0.12 ± 0.064, 1.38 ± 0.56 µM, respectively) [39]. 


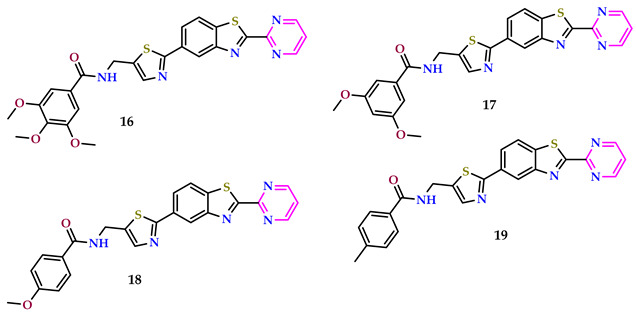


Jame studied the cytotoxicity of thiazolidin-4-one clubbed pyrimidines on HepG2, HCT-116, PC3, MCF-7, and WI38 cells. Cytotoxicity was evaluated by comparing the IC_50_ values to those of the referral drug, 5-fluorouracil. The investigation revealed that hybrid combinations exhibited diverse cytotoxic impacts on cell lines. Notably, hybrids **20** and **21** explained prominent anticancer profiles against MCF-7, with IC_50_ values of 7.53 ± 0.43 as well as 9.17 ± 0.31 μM, respectively. However, **20** showed strong cytotoxicity across four cancer cell lines and exhibited a marked inhibition (IC_50_ = 7.53 ± 0.43 μM) against MCF-7 cell viability, indicating potential selectivity for breast cancer, and lower cytotoxicity in normal WI38 cells, implying a proper selectivity against three other cancer cells, with IC_50_ values of 15.05 ± 0.61, 15.35 ± 0.20, and 16.26 ± 0.42 μM [40].

The IC_50_ values revealed that the benzylidene analogues exhibited strong cytotoxic action, notably against the MCF-7 cancer cell; even the placement of the substituent on the aryl ring influences the relative cytotoxic action and can be regarded as variations in either bioavailability. While hybrid **20** with a 4-methoxyl moiety showed considerable cytotoxicity, the presence of 4-methoxyl suggests more activity than 2-methyl and 3-methyl replacements. 4-methoxy demonstrated greater reactivity, especially against MCF-7.


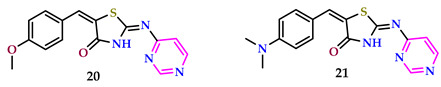


### 2.2. As Antimicrobial Agents

Limaye et al. reported that employing biogenically synthesized single-phase *δ*-MnO_2_ nanoparticles underneath an external ligand-free environment is a sustainable strategy for pharmaceutically important pyrimidine derivatives. Furthermore, investigations were conducted on the growth curve and lowest inhibitory concentration of *δ*-MnO_2_ nanoparticles and pyrimidine derivatives, and compound **22** was tested regarding its antibacterial ability against the Gram-negative bacterium *E. coli* [41].


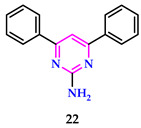


Bryndal et al. produced and tested it for antibacterial efficacy against *E. faecalis* in conjunction with the antineoplastic properties of pyrimidines. The maximum cytotoxicity against RPTEC was seen in **23** (65% at 250 μM after 72 h) and **24** (67% at 250 μM after 72 h). The maximum cytotoxic impact (<100 µM) was reported for **25**, which includes a 3-chlorophenyl analogy in the 5-position. The results for **25** were quite promising and should be useful in anticancer medication research. Synthesized compounds were tested against seven microbial strains (*E. coli*, *S. aureus*, *K. pneumoniae*, *A. baumannii*, *P. aeruginosa*, *E. faecalis*, and *C. albicans*). No MIC or MBC/MFC action was detected at concentrations varying between 0.5 and 256 µg/mL [42].


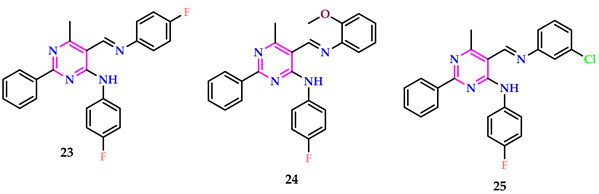


Yao et al. developed various pyrimidine-based complexes. The cytostatic abilities of the eight complexes against HepG-2 and A549 cells were investigated in vitro, indicating that organoantimony derivatives with tolyl groups exhibited superior cytostatic activity. Additionally, bacteriostatic assessments were accomplished to ascertain the inhibitory impacts of the complexes on three powerful plant pathogenic fungi along with a bacterium. The findings demonstrated that the complex of Sb with a methyl position at the third position exhibited strong fungicidal action, but the complex of Sb with a methyl position at the fourth position exhibited better antibacterial activity against MRSA [43].

The effectiveness of organic antimony complexes in biological activities is influenced by several factors, such as the structural characteristics involving oxygen links, the type of organic substituents (like phenyl or tolyl) connected to the antimony atoms, and the function of the ligand, **26**. Among these, antimony complexes featuring oxygen bridge dimers, 3-tolyl substituents, and developed from 4-pyrimidine carboxylic acid exhibited superior anticancer properties compared to other synthetic organic antimony derivatives.


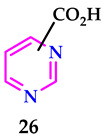


Mahdavi et al. synthesized pyrimidines and studied their antibacterial behavior against *E. coli* and *S. aureus*. Compounds **27** and **28** had the largest inhibition zones for *S. aureus*, while compounds **29** and **30** had the largest inhibition zones for *E. coli* [44].


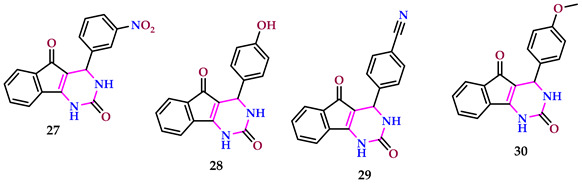


Mor et al. discovered pyrimidines and assessed their in vitro anticancer potential against HCT116, MCF7, and PC3 cell lines, using Camptothecin as an accepted drug reference. They also evaluated their in vitro antimicrobial activity against *E. faecalis*, and *E. coli, S. aureus*, and *S. Typhi*, as well as the fungal strain *R. oryzae*, employing Fluconazole and Tetracycline as standard reference drugs for antifungal and antibacterial testing, respectively. Compounds **31** and **32** exhibited notable inhibition of all cancer cell lines tested, with IC_50_ data ranging from 29.40 ± 0.21 µM-S53.31 ± 0.22 µM, related to the standard medication Camptothecin (0.25 ± 0.09 µM for HCT116, 0.41 ± 0.07 µM for MCF7, and 0.38 ± 0.03 µM for PC3) [45]. 

Compound **33** demonstrated superior performance compared to the standard drug Tetracycline (IC_50_ = 0.0018–0.0128 µM) against two Gram-positive bacterial strains (*S. aureus* and *E. faecalis*) and two Gram-negative bacterial strains (*S. Typhi* and *E. coli*), with IC_50_ values of 0.0252 µM, 0.0029 µM, 0.0062 µM, and 0.0328 µM, respectively, as well as against the fungal strain *R. oryzae* (IC_50_ = 0.0227 µM). Moreover, in the comparison among methyl and phenyl substituents on the pyrimidine ring of pyrazolones, derivatives containing a methyl group exhibited heightened anticancer action against all examined cell types.


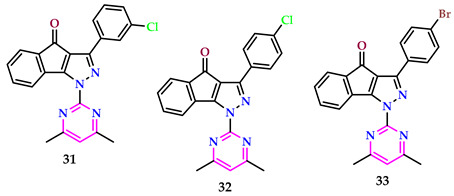


Almakhzoum and Almaqtari reported several pyrimidines and their antibacterial behavior against the investigated bacteria *S. aureus* and *E. coli*, and the fungi *A. flavus* and *A. niger*, which exhibited activity ranging from strong, moderate, and minor for the synthesized pyrimidine compounds. All of the test compounds from **34**, **35**, and **36** showed high action against the bacteria *E. coli* and *S. aureus*, and the fungi *A. flavus* and *A. niger* [46].


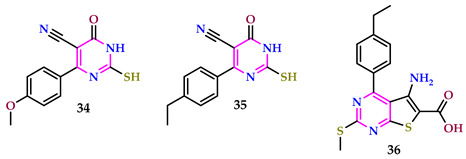


Mamand et al. reported pyrimidines with azo linkages, which were estimated for their antimicrobial action against *S. aureus* and *E. coli* with standard Metronidazole. The findings demonstrated that with increasing concentration, sensitivity also increased, and at a concentration of 800 µg/mL, no bacterial growth was observed. The investigated compounds exhibited enhanced biological action alongside both *S. aureus* and *E. coli*, indicating Gram-positive and Gram-negative bacteria, respectively, at concentrations of 200 μg/mL. Their activity further increased at 400 μg/mL and 600 μg/mL, reaching a point of no growth at 800 μg/mL. Consequently, the synthesized compounds **37–39** displayed considerable biological activity, showcasing notable antimicrobial efficacy against both types of bacteria [47].


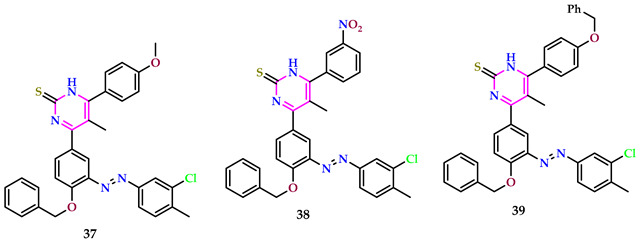


Uysal et al. described a greener method for pyrimidines, and some of the compounds were tested for antibacterial behavior against *S. epidermidis* and *S. aureus* using the reference drugs Ampicillin and Penicillin G. The test outcomes indicated that compounds **40**, **41**, **42**, and **43** exhibited bacteriostatic consequences against *S. aureus*, while compounds **40**, **42**, and **43** displayed such effects against *S. epidermidis*. These conclusions suggest that the existence of allyl, 4-fluorophenyl, and propargyl groups on thiazolo [3,2-*c*]pyrimidines contributes to their antibacterial actions [48].


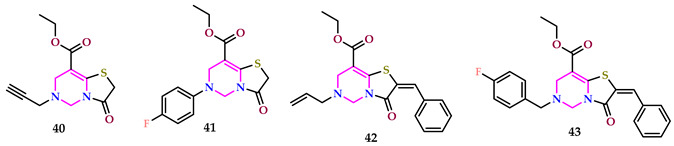


### 2.3. As Anti-Alzheimer’s Agents

Almehizia et al. synthesized pyrazolo-pyrimidine derivatives and screened for various biological potentials using in vitro methods for anti-Alzheimer’s, anti-diabetic, anti-arthritic, antioxidant, and anti-cancer representatives. The antioxidant analysis revealed that compound **44** displayed slightly elevated TAC at 31.27 ± 0.07 mg gallic acid per g, higher Iron-Reducing Power (IRP) at 17.97 ± 0.04 μg mL^−1^, and a lower IC_50_ value against DPPH at 18.33 ± 0.04 μg mL^−1^. Additionally, it exhibited increased inhibitory activity against ABTS at 28.23 ± 0.06% compared to compound **45**, which had TAC of 30.58 ± 0.07 mg gallic acid per g and an IC_50_ value of 17.29 ± 0.04 μg mL^−1^ [49].

The findings from the anti-diabetic evaluation revealed that compound **44** exhibited reduced IC_50_ values against β-glucosidase (5.18 ± 0.01 mg mL^−1^) and α-glucosidase (2.80 ± 0.01 mg mL^−1^), as well as α-amylase (1.80 ± 0.01 mg mL^−1^), when compared to **45**, using the standard drug acarbose as a reference. Compound **44** showed the highest anti-Alzheimer’s effect in terms of AChE activity (16.00 ± 0.04%) with standard drug Donepezil and on proteinase denaturation (17.55 ± 0.04%) (diclofenac sodium as standard) and proteinase action (16.25 ± 0.04%) as an anti-arthritic representative. Compound **44** demonstrates a decreased IC_50_ value of 40.54 μg mL^−1^ for A549 and 29.77 μg mL^−1^ for Caco-2 in terms of cytotoxicity when correlated to the standard drug doxorubicin. The presence of the chloro group at the phenyl ring will be responsible for the enhanced activities in the case of compound **44**.


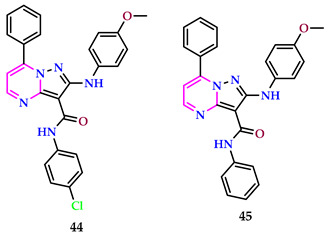


Kahvecioglu et al. described pyrimidines [50]. The inhibitory effect of prepared compounds that could be used to treat Alzheimer’s disease was meticulously tested against the enzymes AChE and BChE. Furthermore, the compounds’ antioxidant activity was investigated because there is a link between the two bioactivities. In ABTS, DPPH, and CUPRAC experiments, compound **46** with a methoxy group outperformed α-tocopherol in terms of antioxidant activity. Compound **47**, with a methoxy group, has the highest antioxidant activity among the thiosemicarbazide-containing compounds when assessed using CUPRAC, ABTS, and DPPH results based on the electron transfer mechanism. This chemical demonstrated higher antioxidant activity than α-TOC and BHA in ABTS and DPPH tests. Compound **46** effectively inhibited both AChE and BChE, with IC_50_ data of 20.15 ± 0.44 µM and 36.42 ± 0.73 µM, respectively. In contrast, the reference medication galantamine had IC_50_ values of 4.82 ± 0.75 µM and 45.54 ± 0.18 µM. Compound **46** has also shown significant antioxidant action.


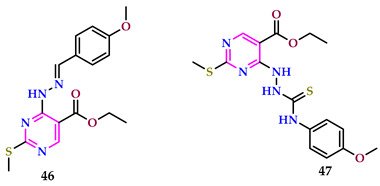


Pant et al. reported pyrimidines and assessed them for anti-Alzheimer’s action. Among the conjugates tested, **48** exhibited superior AChE inhibitory action, with an IC_50_ value of 0.03 ± 0.002 μM alongside AChE, associated with the accepted drug donepezil. In behavioral assessments for memory impairment in mice, compound **48** demonstrated the most powerful effects, drastically ameliorating cognitive deficits at a dosage of 2 mg/kg, surpassing the efficacy of donepezil at the same dosage. Treatment with the substituted pyrimidine **48** aided in restoring normal levels of biochemical intermediaries while also inhibiting reactive oxygen and nitrogen species associated with neuroinflammation mechanisms [51].


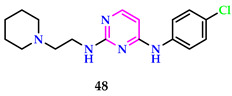


### 2.4. Herbicidal Properties

According to Thakuri et al., the functionalized pyrimidines’ herbicidal properties were evaluated in vitro at various doses against *R. sativus* seeds that had been sterilized. Significant pre-emergent herbicidal activity was demonstrated by the compounds. The findings of the herbicidal activity tests on compounds **49** and **50** showed that they performed better when substituted with chloro (IC_50_ value of 49.82) and methoxy (IC_50_ value of 39.56), but not as well when using normal pendimethalin (IC_50_ value of 56.52). The compounds performed well for in vitro and in silico experiments, offering plenty of room for additional adjustments and research into the potential of phenyl pyrimidines as a strong herbicide [52].


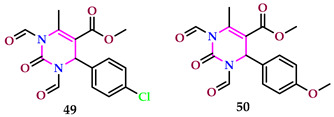


Luo et al. conducted the first screening of pyrimido-triazolo-pyrimidines and their herbicidal properties using both Petri dish assessments and pot testing in a glasshouse. Compound **51** exhibited effective post-emergence herbicidal action against *A. theophrasti*, *A. spinosus*, *C. album*, *Echinochloa crusgalli*, *D. sanguinalis*, and *S. viridis* at a scale of 375 g/ha, comparable to the positive controller Flumetsulam. Enzymatic bioassays showed that compounds **51**, **52**, and **53** had significant inhibitory activity against ALS, with inhibition rates of 39.6%, 39.1%, and 40.9%, respectively, like Flumetsulam’s rate (43.5%). SAR studies revealed that compounds with C_5_H_11_ exhibited higher herbicidal efficacy compared to long-chain compounds. Substituents 4-F, 3-CF_3_, 4-Cl, and 2,4-di-Cl did not notably affect herbicidal action. Additionally, the presence of benzoyl did not enhance herbicidal efficacy [53].


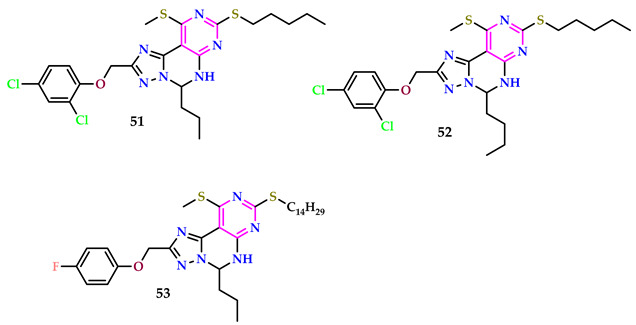


### 2.5. As Antioxidant

Abouzayed et al. identified a few pyrimidine complexes based on bioactive chelates and assessed them for cytotoxicity against MCF-7 and Hep-G2 cells. All of the analyzed chelates, except Sm(III) chelate, were more cytotoxic to cancer cells than the parent ligand, **54**. Except for Zn(II) chelate, which showed activity on Hep-G2 instead of MCF-7, each metal chelate’s effects on the two cells were almost identical. According to the Zn(II) complex’s trend, Hep-G2 was the target of more selectivity than MCF-7. Compound 54’s association with certain metal ions enhanced its cytotoxic action. Compound **54** (IC_50_ = 42 μg/mL) was not as powerful an antioxidant as any of the investigated chelates, particularly Zn(II) (IC_50_ = 20 μg/mL) and Co(II) (IC_50_ = 28 μg/mL) [54].


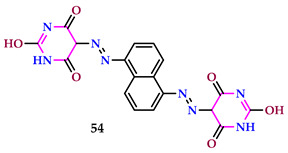


Alzahrani et al. exploited sequences of spiro pyrrolo-pyrimidines and investigated them against DPPH while evaluating their ability to inhibit COX-1 and COX-2 enzymes. All complexes displayed substantial anti-inflammatory action, inhibiting both COX-1 and 2 enzymes with an SI higher than that of celecoxib, a referral drug. Among them, compounds **55** and **56** emerged as the utmost effective and selective COX-2 inhibitors, with SIs of 175 and 129.21, respectively, compared to celecoxib’s index of 31.52. Notably, candidate **57** exhibited highly promising anti-inflammatory activity, with an IC_50_ value of 6.00, whereas celecoxib had an IC_50_ of 14.50. Additionally, compound **55** demonstrated strong antioxidant activity, with an IC_50_ value of 33.0 μg mL^−1^, compared to the standard ascorbic acid’s IC_50_ of 4.08 μg mL^−1^, along with DPPH scavenging percentages of 86.1% and 97%, respectively [55].


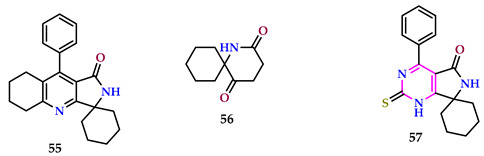


Bouguessa et al. developed pyrimido-pyrimidine and pyrimido-purines, as well as testing their antibacterial profiles against Gram-positive (*S. aureus*) and Gram-negative (*E. coli*) bacteria. Compound **58** showed modest efficacy against *E. coli* at 25 μg/mL, with an inhibition diameter of 12 mm. It inhibited *S. aureus* with inhibition diameters of 13 mm, 10 mm, and 8 mm at concentrations of 25 μg/mL, 12.5 μg/mL, and 6.25 μg/mL, respectively. Additionally, they underwent in vitro antioxidant action testing via DPPH and ABTS radical scavenging assays, where compounds **58** and **59** demonstrated moderate effectiveness in scavenging radicals [56].

The findings indicated that compound **59** exhibited the most potent reducing power, with an IC_50_ value of 22.02 μg/mL in the DPPH and 90.72 μg/mL in the ABTS examinations. This could be attributed to the absence of an amide substitution attached to the adenine nucleus. Compound **58** displayed a scavenging capacity comparable to compound **59**, with measurements of 27.11 μg/mL in DPPH and 97.86 μg/mL in ABTS.


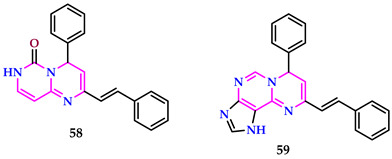


Abu-Hashem et al. synthesized some pyrimidines, and their anticancer activities against MCF-7 and RPE-1, as well as their antioxidant activities, were studied. The research unveiled significant antiproliferative activity of certain compounds against MCF-7 and RPE1 carcinoma cell lines, with IC_50_ values ranging from 6.2 to 15.1 μM for MCF-7 and from 17.5 to 26.4 μM for RPE-1 cells. Compounds **60**, **61**, **62**, and **63** demonstrated high efficacy in targeting carcinoma cell lines, remarkably MCF-7 and RPE-1. Furthermore, these compounds possess diverse functional groups and moieties, contributing to their enhanced cytotoxicity matched to the drug, Doxorubicin. The results suggest that compounds **63**, **62**, **61**, and **60** exhibit anticancer action alongside antioxidant results, as evidenced by their DPPH inhibition [57].


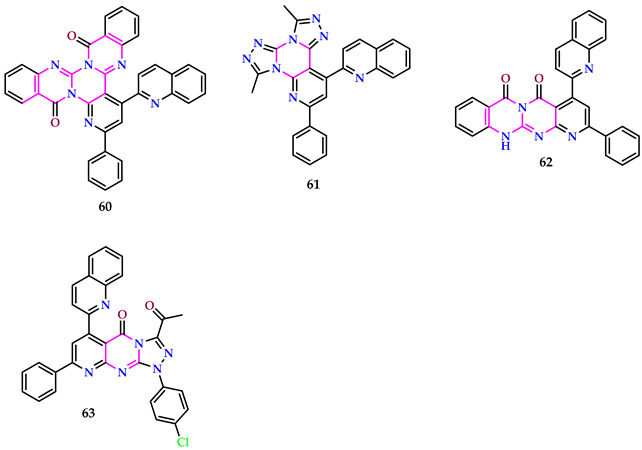


Sivanandhan and Parasuraman discovered pyridopyrimidines. Compound **64** demonstrated the strongest antibacterial activity. Chloramphenicol was used as a standard drug. Regarding antifungal action, the compounds containing electron-drawing groups pointed to an improved zone of inhibition, except that *C. albicans* fluconazole was used as a standard. Compound **65** demonstrated the strongest activity against *A. niger*, *C. albicans*, and *A. parasiticus*. However, compound **64** had the highest activity against *A. fumigatus*. Compounds (**66**, **67**, and **68**) containing electron-donating groups positioned parallel to the phenyl nucleus displayed heightened antioxidant action. The compounds with electron-donating groups showcased outstanding capability in scavenging free radicals [58].

Compounds **64** and **65**, featuring electron-withdrawing groups, demonstrated enhanced anticancer activity. Due to its potent anticancer effects, compound **64** was preferred for cytotoxicity evaluation against the L929 mouse fibroblast cell line based on its IC_50_ values. When compared to their respective standards, halogen-substituted compounds demonstrated greater antibacterial and anticancer action. Based on the anticancer activity IC_50_ value, **64** was examined for apoptosis using the AO/EB staining mode. The data pointed to early apoptosis in the MCF-7 cell line.


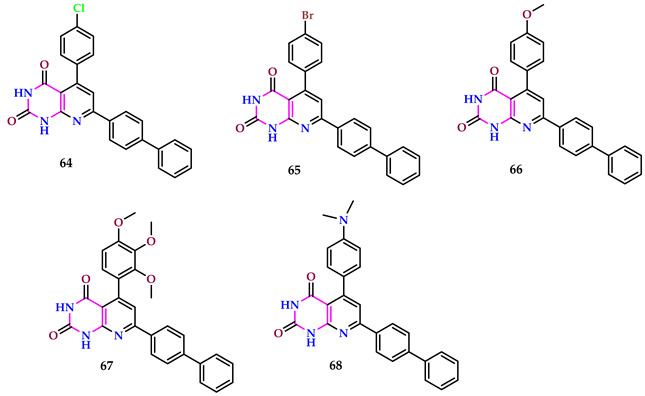


### 2.6. Use of Pyrimidine Core as Insecticide

Abbass et al. produced spiro pyrimidines and tested their toxicological efficiency and biological effects on *C. pipiens* L. larvae. The toxicity of produced compounds differed significantly against *C. pipiens* larvae. The most effective compounds were **69**, **70**, and **71**, with LC_50_ values of 12.43, 16.29, and 21.73 µg/mL, respectively, and exhibited minimal to no harmful effects. Apart from their notable effectiveness against mosquito larvae, compounds **69** and **70** inhibited adult emergence at a concentration of 100 µg/mL. Compounds **71**, **69**, and **70** not only hindered the advancement and growth of *C. pipiens* larvae but likewise induced numerous morphological abnormalities, attributed to the enhanced activity resulting from the fused aromatic ring. The cyclocondensation of spiro pyrimidine leads to combined pyrimidopyrimidine, which incorporates an amide C=O group, further enhancing the action of compounds **71** and **69** [59].


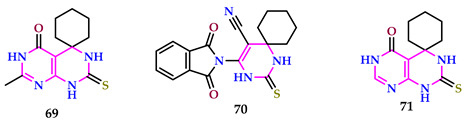


El-Lateef et al. reported indole clubbed heterocycles. The LC_50_ values of compound **72** (pyrimidine ring linked to a pyran ring) are 0.44 ppm and have the highest insecticidal activity against *C. aphids* (nymphs). LC_50_ values of compound **72** are 1.00 ppm and have the highest insecticidal activity against C. aphids (adults) [60]. Compound **72** exhibited the highest impact against *C. neoformans* at a dosage of 20 mg/mL, with a 96.81% inhibition zone. Compound **72** had a high impact on *S. racemosum*, with 95%.


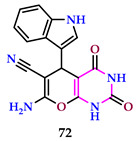


### 2.7. Use of Pyrimidine Core as Anti-Inflammatory

Sayed et al. investigated indole–pyrimidine hybrids and assessed their anti-inflammatory activity. The incorporation of an indole moiety enhances the structural diversity of these compounds, potentially exerting a significant influence on their biological activities. Compounds **73**, **74**, **75**, and **76** demonstrated notable activity as potential candidates, exhibiting 43.17%, 40.91%, 36.35%, and 43.17% inhibition after 4 h in paw edema, and 26.67%, 35.56%, 26.67%, and 31.10% inhibition after 5 h, respectively. These values were compared to indomethacin, a standard drug, which showed 47.72% and 42.22% inhibition at 4 and 5 h, respectively. Furthermore, compounds **73**, **74**, **75**, and **76** demonstrated higher anti-inflammatory efficacy than indomethacin. The elevated activities in cases **73**, **74**, **75**, and **76** will be caused by the presence of oxygen at the pyrimidine ring, the existence of a chloro group at the pyrimidine nucleus, the presence of sulfur at the pyrimidine ring, and the presence of an amido side chain [61].


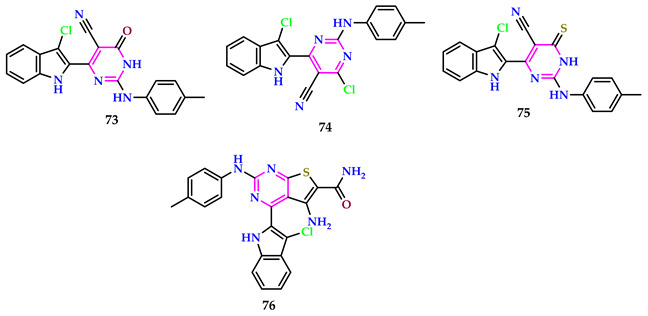


Bafail and Samman reported a variety of poly-fused pyrimidine derivatives [62]. Compared to Benzotropene^®^ (1.00 ± 0.02), compounds **77** and **78** showed significant anti-parkinsonian activity (0.76 ± 0.01, 0.82 ± 0.02 inhibition). Compound **78** had comparable anti-inflammatory activity (62.3 ± 1.1) to the reference medication, Indomethacin^®^ (65.4 ± 1.2). Compound **78** demonstrated no significant ulcerogenic activity when compared to the control group. Compound **77** strongly inhibits *S. aureus*, *E. coli*, *K. pneumoniae*, *B. subtilis*, and *C. albicans* (*p* < 0.05). Compound **79** successfully inhibited both *S. aureus* and *E. coli* (*p* < 0.05). Compounds **80** and **81** strongly inhibited *S. aureus*, *E. coli*, *K. pneumoniae*, *B. subtilis*, *C. albicans*, and *A. fumigates* compared to ciprofloxacin and ketoconazole (*p* < 0.05). Compound **82** had considerably higher analgesic activity (1.12 ± 0.01) than Valdecoxib^®^ (1.00 ± 0.01) after 60 min.

Compounds **77** and **78** had substantial hypoglycemic effects (80.7 ± 2.2, 82.2 ± 2.4 mg/dL, respectively) after 24 h (*p* < 0.05). Compounds **77** and **78** have significant hypoglycemic effects (81.2 ± 2.3, 84.4 ± 2.4 mg/dL) after 72 and 120 h (82.6 ± 2.5, 87.5 ± 2.2 mg/dL, respectively). As compared with pioglitazone^®^ (100.0 ± 2.2 mg/dL), compounds **77** and **78** showed considerably stronger hypoglycemic action (81.3 ± 1.7 and 85.1 ± 1.2 mg/dL, respectively) compared to pioglitazone^®^ (100 ± 2.1 mg/dL).

Compounds **80** and **81** outperformed doxorubicin^®^ (6.52 mol/L) regarding anticancer action against HT-29, with IC_50_ values of 6.38 and 7.82 mol/L, respectively. Compound **80** outperformed doxorubicin^®^ (6.83 mol/L) against DU145.


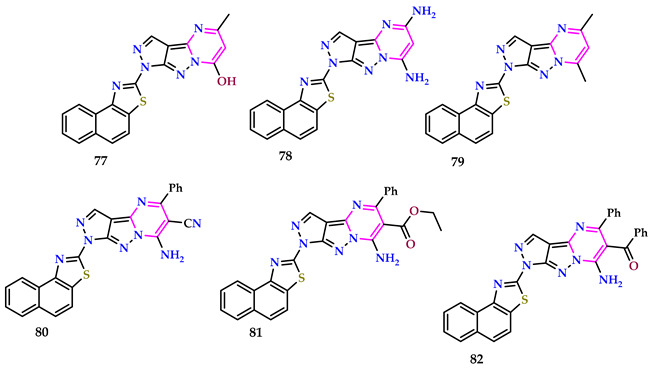


### 2.8. Use of Pyrimidine Core as Anti-Diabetic

Kamat et al. analyzed pyrimidines for α-amylase and α-glucosidase activity. Compound **83** had a much lower IC_50_ value for α-amylase (1.08 ± 0.42 mg/mL) compared to normal Acarbose (1.42 ± 0.46 mg/mL). Compound **83** has an IC_50_ of 92.64 ± 0.42%, and compound **84** has an IC_50_ of 89.05 ± 0.16%, while normal Acarbose showed 70.61 ± 0.46% inhibition of α-amylase at 2 mg/mL. Compounds **83** and **84** demonstrated the most powerful inhibitory action against α-glucosidase, with IC_50_ values of 1.16 ± 0.72 mg/mL and 1.1 ± 0.68 mg/mL, respectively. These values surpassed those of the reference medication acarbose, which had an IC_50_ value of 1.42 ± 0.46 mg/mL. At a concentration of 2 mg/mL, compounds **83** and **84** demonstrated α-glucosidase inhibitory activities of 90.94 ± 0.68% and 85.92 ± 0.72%, respectively, while standard acarbose inhibited α-glucosidase by 69.89 ± 0.61% [63].


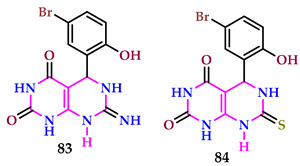


Mallidi et al. reported pyrimidine-based carbocyclic nucleosides [64]. Compounds **85** and **86** demonstrated encouraging IC_50_ values against α-glucosidase, measuring 43.292 nmol and 48.638 nmol, correspondingly. Additionally, compounds **86** and **87** showed enhanced antimicrobial action against *B. cereus*, with zone of inhibition values of 2.2 ± 0.25 mm and 1.4 ± 0.1 mm, respectively, at a concentration of 100 μL. Furthermore, compound **86** displayed a moderate zone of inhibition of 1.2 ± 0.15 mm against *E. coli* at 100 μL.


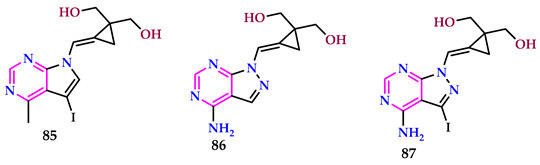


### 2.9. Use of Pyrimidine Core as Antiviral

Gallo et al. conducted a low-throughput antiviral investigation, identifying compound **88** as an effective inhibitor of ZIKV and DENV-2, with EC_50_ data of 2.4 μM and 1.4 μM, respectively. Compound **89** exhibited lower toxicity compared to similar compounds. Compound **90** demonstrated significant antiviral action against both ZIKV and DENV-2. Even with its toxicity, **90** exhibited the highest SI [65].


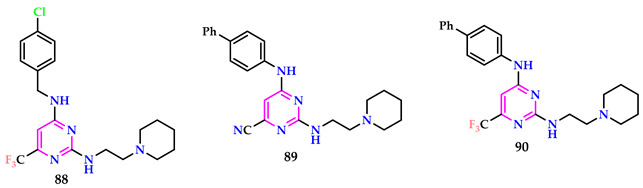


Song et al. reported pyrimidines containing long chains. Following 48 h of treatment, compound **91** markedly suppressed cell growth and viability in PK-15 cells (** *p* = 0.002869). Compound **92** generated a substantial G1-phase arrest in PK-15 cells linked to MOCK (* *p* = 0.03138) post-treatment of 24 h. Compound **93,** after 48 h, had a changed appearance on the cell cycle. In PK-15 cells, the compound **93**-treated group had a significantly higher cell apoptosis rate than the control group (** *p* = 0.007938). Except for compound **93**, these tiny molecules can initiate NF-κB promoters [66].

Compounds **94**, **95**, **92**, and **96** exhibited superior antiviral efficacies compared to others, with IC_50_ values of 10.06, 1.38, 1.93, and 8.3 µM, respectively. These drugs significantly suppressed viral replication when cells were infected with PRV-GFP (MOI = 0.1). Their IC_50_ values for PRV-GFP were 29.05, 0.15, 0.13, and 0.095 µM, respectively. Apilimod, a positive control antiviral drug, showed IC_50_ values of 0.66 µM for PRV-GFP and 2.16 µM for VSV-GFP. In vivo studies demonstrated that compounds **94**, **92**, **95**, and **96** effectively protected mice from PRV-QXX infection, as evidenced by reduced viral growth in lung tissues. Compound **95** displayed the most powerful antiviral action, lowest virus load, and minimal tissue damage.


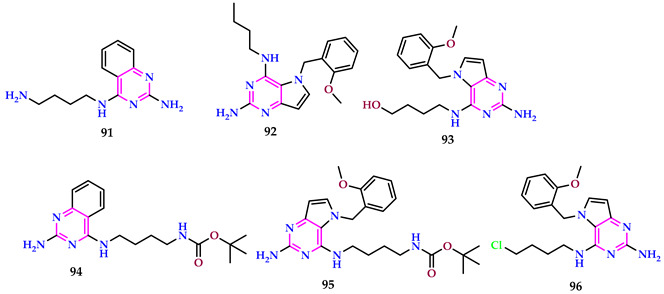


### 2.10. Use of Pyrimidine Core as Antitubercular

Cele [67] et al. reported quinoline-pyrazolopyrimidines, with 4-methylpiperidine compounds (**97**) and 4-trifluoromethoxy (**98**) groups, respectively, demonstrating the greatest securing α-glucosidase inhibition behavior, with IC_50_ values of 46.70 and 40.84 μM, related to the referral inhibitor, acarbose (IC_50_ = 51.73 μM). SAR exploration suggested that the pendants, i.e., cyclic secondary amine and 4-phenyl substitutions, describe the inconsistent enzyme inhibition. Compounds **98** (IC_50_ = 40.84 μM) and **99** (IC_50_ = 45.99 μM) bearing electron-withdrawing substituents were found to be more potent inhibitors. Antioxidant action further revealed that *N*-methylpiperazine compounds (**100**) and (**101**) with the *N*-ethylpiperazine ring, respectively, have decent DPPH scavenging capabilities, with IC_50_ = 0.18, 0.93, and 0.58 mM, as related to ascorbic acid (IC_50_ value of 0.05 mM), while the finest DPPH scavenger is the NO_2_^−^ attached compound (IC_50_ value of 0.08 mM). Also, in the *N*-(2-hydroxyethyl)piperazine substituted moiety, **102** appeared as the leading NO radical scavenger, with IC_50_ value of 0.28 mM. Compound **99** emerged as the best DPPH scavenger, with an IC_50_ value of 0.08 mM, akin to the reference antioxidant, ascorbic acid (IC_50_ of 0.05 mM).


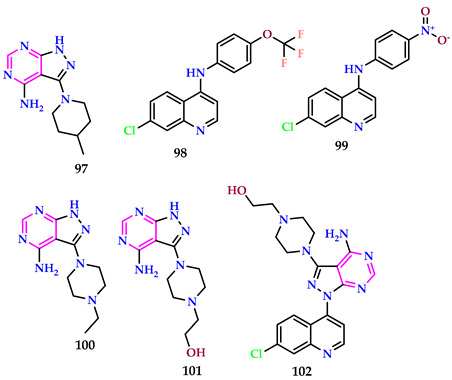


Raghu et al. reported thiazolidinedione-containing pyrimidines [68]. Active compounds **103**, **105**, and **104** displayed 1.85, 1.38, and 1.15 times the action of streptomycin, counter to *S. aureus*, with MIC values of 6.4, 8.6, and 10.3 µM, respectively. Compound **103**, featuring a trifluoromethyl group attached to the phenyl nucleus at the fourth place of the thiazolidinedione part, **105** (with a 3,4,5-trifluoro group), along with compound **104** (with a 3,5-difluoro group), and exhibited 2.14, 1.50, and 1.05, times the action of linezolid against the MRSA strain, with MIC values of 10.8, 21.9, and 15.4 µM, respectively. Furthermore, compounds **103**, **104**, and **105** outperformed the reference drugs in terms of antibacterial effectiveness against all tested bacterial and fungal strains. Compounds **103**, **104**, and **105** had significant anti-tubercular efficacy among all compounds tested. Compound **103** is the most effective, 1.59 times more so than the reference medication isoniazid. Among the analyzed derivatives, compounds **103**, **105**, and **104** exhibited the greatest SI values of 227.27, 156.25, and 70.42, respectively. Isoniazid, with an SI value of 142.86, presents a promising prospect for further exploration of derivatives based on compounds **103**, **104**, and **105**.


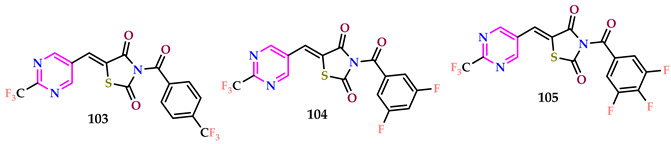


### 2.11. DNA Binding Studies

Abdelwahab et al. reported pyrimidines, and the well diffusion method was utilized to analyze the antibacterial properties of the synthesized compounds against various bacterial strains, and it was discovered that **106** was the most effective [69]. Among the produced compounds, the analogue (**106**) with an electron-drawing group (chloro substitution) was effective against the Gram-negative bacteria tested. The results support the chemical **106** affinity for CT-DNA, as they show that the absorbance of the *N*-arylacetamide conjugative **106** decreases as the concentration of CT-DNA rises. The interaction of **106** with DNA resulted in a notable hypochromism (33%). This contact also resulted in a small redshift (~5 nm). Absorption spectroscopy revealed that compound **106** had a high potential for DNA binding, with an intrinsic binding constant value of 2.64 × 10^4^ M^−1^.


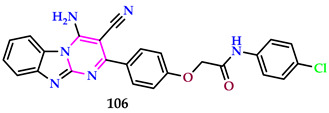


Dominguez et al. assessed the characteristics of PPRHs targeting the SARS-CoV-2 genome. Numerous PPRHs were produced to aim at different polypyrimidine locations in the viral genome. The binding affinities of these PPRHs varied depending on their length and GC contents. The quantity and placement of pyrimidine interruptions about the 4 T loop of PPRHs were identified as crucial determinants of their binding attraction with the correct target. Likewise, these elements were observed to impact the intramolecular and intermolecular equilibria of PPRHs both individually and when paired with their respective directs, emphasizing the polymorphic environment of these arrangements [70].

### 2.12. Auxin-like and Cytokinin-like Effect

Anatolyivna et al. studied the regulatory impact of thienopyrimidine products on the vegetative growth and photosynthesis of the wheat variety Svitlana (*T. aestivum* L.). They compared the regulatory effects of newly synthesized thienopyrimidine derivatives with those of auxin IAA and synthetic plant growth controllers Kamethur and Methyur. The morphometric and biochemical considerations of wheat plants were regulated similarly or more effectively by the new synthetic thienopyrimidine derivatives compared to auxin IAA or synthetic plant growth controllers Kamethur and Methyur [71].

The newly synthesized thienopyrimidine derivatives (compounds **107**, **108**, **109**, **110**, **111**, and **112**) exhibited a greater growth-regulating impact compared to auxin IAA or synthetic plant growth regulators Methyur and Kamethur, as indicated by morphometric strictures of wheat plants, including the average length of shoots and roots (in millimeters) and the average biomass of 10 plants (in grams).


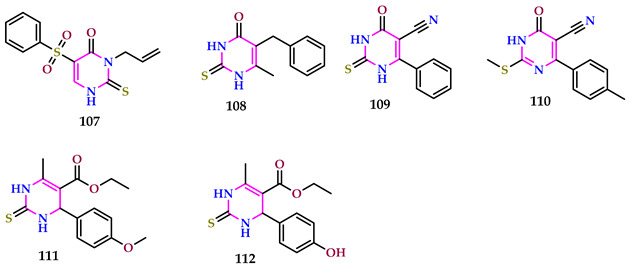


## 3. Conclusions

This review discusses the most recent advances in pyrimidines with remarkable biological characteristics. Pyrimidines are known for their high biological efficacy, and much research has been conducted to investigate the relationship between their structural properties and biological activities. Pyrimidines have demonstrated remarkable adaptability in targeting a variety of molecular targets in the medical profession. This review aims to be a significant resource for future research and the creation of novel compounds that boost biological activity. This review helps medicinal chemists design and develop clinical candidates with greater selectivity and potency by offering a complete examination of structure–activity correlations. The findings of this study are likely to contribute significantly to ongoing research efforts and may facilitate the discovery of novel pyrimidines with therapeutic potential, resulting in the development of safer and more effective drugs.

## Figures and Tables

**Table 1:** IC_50_ values of pyrimidine-pyrazine-oxazoles [37].

Compound	SiHa (µM)	A549 (µM)	MCF-7 (µM)	Colo-205 (µM)
12	0.03 ± 0.0076	0.01 ± 0.0054	0.12 ± 0.055	0.34 ± 0.033
13	0.071 ± 0.0048	0.09 ± 0.0018	0.011 ± 0.0067	0.16 ± 0.025

Sources: Sabita et al.

## Data Availability

Data sharing is not applicable.
